# CT-based morphological study of the pelvis in patients with gluteal muscle contracture

**DOI:** 10.1186/s13018-023-03564-4

**Published:** 2023-02-08

**Authors:** Yikun Zhao, Xueping Dong, Zhen Zhao, Maojiang Lv, Shun Li, Xintao Zhang

**Affiliations:** 1 Peking University Shenzhen Hospital, Clinical College of Anhui Medical University, Shenzhen, China; 2 Department of Sports Medicine and Rehabilitation, Peking University Shenzhen Hospital, Shenzhen, China

**Keywords:** Gluteal muscle contracture, Pelvic rotation, Acetabular coverage angle, Acetabular retroversion

## Abstract

**Background:**

In the clinic, gluteal muscle contracture (GMC) causes pelvic structural changes, including acetabular retroversion. However, its causes and forms are not well understood. This study aimed to investigate and analyse the clinical significance of pelvic structural differences between GMC patients and healthy individuals.

**Methods:**

As the GMC group, we identified 100 GMC patients who received treatment and met the inclusion criteria between January 2019 and January 2020. Control subjects were drawn from the hospital’s emergency trauma patients who had no history of pelvic or hip joint disease. All subjects underwent CT scans to measure their pelvic rotation, including the superior iliac angle (SIA), inferior iliac angle (IIA), and ischiopubic angle (IPA), and acetabular coverage, which includes anterior acetabular sector angle (AASA), posterior acetabular sector angle (PASA), horizontal acetabular sector angle (HASA), and superior acetabular sector angle (SASA).

**Results:**

The SIA, IIA, IPA, and PASA of the GMC group were considerably smaller than those of the control group, while the AASA of the GMC group was higher, indicating a statistically significant difference (*P* < 0.05). The HASA and SASA of the GMC group, on the other hand, were not considerably different from those of the control group. The angles in the GMC group were relativized as follows: The HASA had a positive correlation with the AASA and PASA (*r* = 0.750, *P* < 0.01; *r* = 0.749, *P* < 0.01); the SASA had a positive correlation with the AASA, PASA, and HASA (*r* = 0.555, *P* < 0.01; *r* = 0.273, *P* < 0.01; *r* = 0.552,* P* < 0.01); the AASA had a negative correlation with the SIA, IIA and IPA (*r* = − 0.355, *P* < 0.01; *r* = − 0.551, *P* < 0.01; *r* = − 0.30, *P* < 0.01); the PASA had a positive correlation with the IIA (*r* = 0.315, *P* < 0.01) and had no correlation with the SIA and IPA (*P* > 0.05); and the IIA had a positive correlation with both the SIA and IPA (*r* = 0.664,* P* < 0.01; *r* = 0.465,* P* < 0.01).

**Conclusion:**

Individuals with GMC have an abnormal pelvic morphology, with acetabular retroversion caused by ilial rotation rather than dysplasia of the acetabular wall.

## Background

Gluteal muscle contracture (GMC) is a clinical syndrome characterized by gluteal muscle and fascia fibre degeneration, necrosis, and fibrosis. This change results in limited adduction and internal rotation of the hip joint, showing malformed signs of abduction and external rotation [1]. The main clinical manifestations of GMC are "duck step" gait, inability to sit with crossed legs, inability to squat with adduction of the hip, snapping hip, and other restrictive conditions. At the same time, the long-term contracture state will cause a series of changes in bone morphology, which can seriously influence the patients' quality of life and mental health. After Valderrama’s first revelation in 1970, GMC began to emerge in reports from domestic to international sources. GMC is most prevalent in underdeveloped countries, with rates ranging from 1 to 2.5% [2–4].

Thanks to advances in imaging technology, the typical morphological malformations of the pelvis caused by GMC, such as pelvic tilt, increased CE angle, shallow acetabular depth, hip exostosis, and the ensuing pseudo-isometric bilateral lower extremities, have garnered widespread attention [5, 6]. Nevertheless, studies using CT scans to observe the morphological transformation of the pelvis are relatively rare. Researchers, including You et al. [7], utilized CT to analyse the equatorial-edge angle of 23 GMC patients, discovering that their angles were relatively smaller than those of normal individuals, indicating a retroverted acetabulum. Acetabular retroversion is a common clinical change in bone morphology that leads to changes in the pressure between the acetabulum and the femoral head, resulting in the occurrence of hip osteoarthritis, which is common in hip dysplasia and Perthes’ disease [8].

To date, there has been no report on large-scale CT scan studies to examine the rotational malformation of the GMC pelvis. Dysplasia of the front and rear acetabular walls, as well as pelvic rotation, can promote acetabular retroversion [9]. However, the cause of acetabular retroversion of the GMC has yet to be found. Therefore, the aim of this study was to further clarify the form of GMC acetabular retroversion and pelvic structural alteration by measuring and identifying the difference in pelvic rotation and acetabular coverage angles between GMC patients and normal people.

## Materials and methods

A retrospective design was adopted for this study, and the present study was approved by Peking University Shenzhen Hospital Ethical Review Approval.

### Subjects

Clinical data from GMC patients with bilateral gluteal muscles contracture who underwent CT scans at Peking University Shenzhen Hospital between January 2019 and January 2020 were retrospectively collected. The inclusion criteria were as follows: (1) confirmed cases of GMC by clinical symptoms, physical examination, or arthroscopy, and the affected gluteus muscle and the severity of bilateral contractures in individuals were the same; (2) individuals with no other diseases influencing pelvic structure or hip joint structure, including coxa plana, acetabular dysplasia, slipped capital femoral epiphysis (SCFE), or hip infection; and (3) no abnormal posture such as a pelvic tilt of more than 3°, unintentional pelvic anteversion or retroversion (distance between the sacral joint and the symphysis pubis of less than 0.5 cm or greater than 6.5 cm) during shooting [10]. One hundred GMC patients (100 hips) were eligible for inclusion in this study, including 44 males and 56 females, ranging in age from 19 to 43 years, with a mean age of 33 years and a BMI of 22.5 kg/m^2^.

Additionally, during the same time period, we collected emergency trauma patients with no evidence of a pelvic or hip joint fracture under a CT scan. The inclusion criteria were as follows: (1) no evidence of pelvic or acetabular joint break on a CT scan; (2) no medical history of incidences affecting pelvic or acetabular structures or history of trauma operation; and (3) no history of gluteal muscle injection in childhood or similar symptoms of GMC. The subjects included 47 males and 53 females ranging in age between 18 and 48 years, with a mean age of 35 years and a BMI of 23.1 kg/m^2^. There was no statistically significant difference between the two groups in terms of sex composition, age, or BMI.

### Measurements

In this study, a Philips Brilliance 64-layer spiral CT machine was used. Patients in both groups were positioned lying flat on the CT examination bed, feet together, with a neutral lower limb position and a straight trunk free of lateral obliquity. The scan incorporated the following areas: the top margin of the pelvis to the lesser trochanter of the femur. The scanning parameters were 120 kV spherical tube voltage, 100 mA current, 1.5 mm layer thickness, and 1 mm layer distance, and the below-described indicators were assessed.

Pelvic rotation angles were measured through the following indicators [11]: ① superior iliac angle (SIA): the angle formed by the line connecting the medial edge of the anterior superior iliac spine and the anterior edge of the sacroiliac joint and the horizontal line on the axis; ② inferior iliac angle (IIA): the angle formed by the line connecting the vertex of the anterior inferior iliac spine and the vertex of the posterior iliac bone in the same plane and the horizontal line on the axis; ③ ischiopubic angle (IPA): the projection angle formed by intersecting the line connecting the pubic symphysis and the sciatic spine on the superimposed plane with the sagittal line on the axial plane. The superimposed plane portrayed a cross section of the sciatic spine and the superior border of the pubic symphysis.

**Fig. 1:**
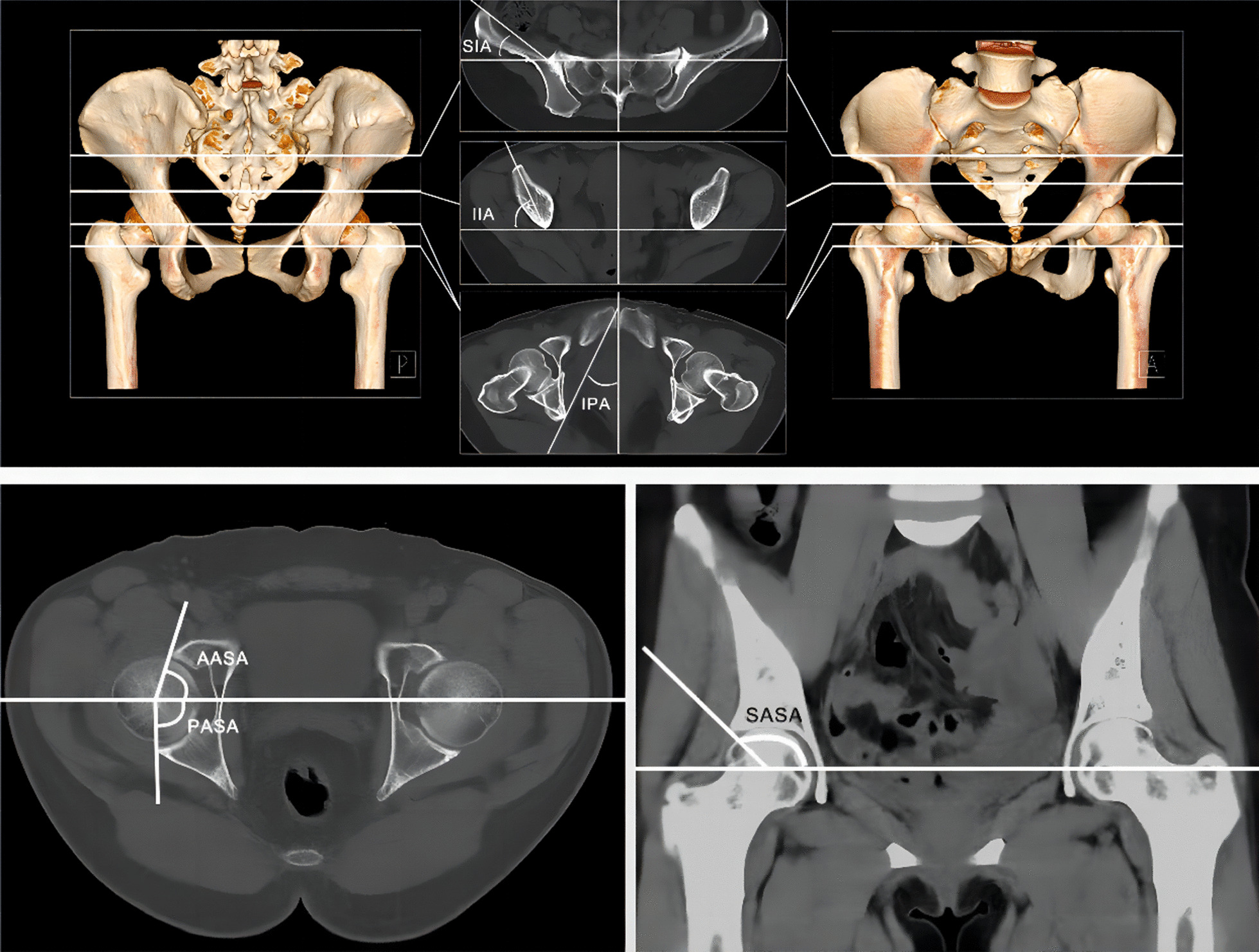
Schematic diagram of the imaging index measurement

We measured the acetabular coverage angle according to the specified methods [12]. In the cross-sectional plane, we chose the level of the femoral head with the greatest cross-sectional area, drew a horizontal line through the centre points of the two femoral heads as the baseline, and connected the anterior and posterior edges of the acetabulum through the centre point again. Correspondingly, the angle between this line and the baseline referred to the acetabular coverage angle, including ④ the anterior acetabular sector angle (AASA) and ⑤ the posterior acetabular sector angle (PASA); likewise, if we were to connect the superior acetabular rim to the centre of the femoral head on the coronal plane, we obtained ⑥ the superior acetabular sector angle (SASA) and ⑦ the horizontal acetabular sector angle (HASA) = AASA + PASA (Fig. 1). Since the extent of gluteus contracture was nearly the same on either side, the pelvic and acetabular angles were measured and compared on only one side in the GMC and control groups.

To evaluate the accuracy and reliability of the measurements, 10 subjects were identified at random from the GMC and control groups. The same observer (A) measured the pelvic angle of these 20 subjects separately at one-week intervals to compare and analyse the intragroup variation in the data; at the same time, the observers (B and C) measured the aforementioned indices separately to compare and analyse the intergroup variation in the data.

### Statistical methods

Power analysis was used to determine the sample size ($\alpha$ err prob = 5%, $1-\beta$ err prob = 80%). For statistical analysis, SPSS 20.0 statistical software was utilized, and the Kolmogorov–Smirnov test was used to determine whether the data were normally distributed. For measurement data, normally distributed data were characterized as the mean ± SD, but nonnormally distributed data were reported as the median (p25, p75). The t test and Pearson's correlation analysis were performed to compare normally distributed data across groups using independent samples. For nonnormal distributed data, the Mann‒Whitney U test and Spearman correlation analysis were employed to compare groups. Differences were considered statistically significant at *P* < 0.05.

In the data reliability analysis, the current study employed the intraclass correlation coefficient (ICC) to quantify intragroup and intergroup variability. “ICC > 0.75” indicated great reliability, and “ICC < 0.4” indicated poor reliability; the higher the value, the better the consistency.

## Results

The data between the groups were normally distributed. The SIA, IIA, IPA, and PASA in the GMC group were lower than those in the control group, whereas the AASA was larger than that in the control group. The differences were all statistically significant (*P* < 0.05), and there was no significant difference between the GMC group and the control group in terms of HASA and SASA (Table 1).

**Table 1:** CT measurement values in the GMC and control groups*

	AASA	PASA	SASA	HASA	SIA	IIA	IPA
Control group	61.4 ± 6.8	100.7 ± 8.7	125.8 ± 6.1	162.1 ± 12.8	50.5 ± 5.3	68.5 ± 3.9	27.0 ± 3.1
Patient group	69.1 ± 9.1	95.6 ± 9.1	125.2 ± 6.1	164.7 ± 13.7	43.0 ± 7.5	62.2 ± 6.2	23.1 ± 3.2
*t*	6.783	− 4.081	− 0.611	1.362	− 8.17	− 8.628	− 8.851
*P*	< 0.05	< 0.05	0.542	0.175	< 0.05	< 0.05	< 0.05

*Unit: °; Values are presented as the mean ± SD

The angles in the GMC group had the following correlations. Among acetabular coverage angles, HASA had a positive correlation with AASA and PASA (*r* = 0.750, *P* < 0.01; *r* = 0.749, *P* < 0.01), while SASA had a positive correlation with AASA, PASA, and HASA (*r* = 0.555, *P* < 0.01; *r* = 0.273, *P* < 0.01; *r* = 0.552, *P* < 0.01). Between acetabular coverage angles and pelvic rotation angles, AASA had a negative correlation with SIA, IIA, and IPA (*r* = − 0.355, *P* < 0.01; *r* = − 0.551, *P* < 0.01; *r* = − 0.30, *P* < 0.01); PASA had a positive correlation with IIA (*r* = 0.315, *P* < 0.01) and had no correlation with SIA and IPA (*P* > 0.05). Among the pelvic rotation angles, IIA had a positive correlation with SIA and IPA (*r* = 0.664, *P* < 0.01; *r* = 0.465, *P* < 0.01) (Table 2).

**Table 2:** Correlation of each parameter in the GMC group

	AASA	PASA	HASA	SASA	IPA	SIA	IIA
AASA	1	0.124	0.750**	0.555**	− 0.300**	− 0.355**	− 0.551**
PASA		1	0.749**	0.273**	0.104	0.166	0.315**
HASA			1	0.552**	− 0.131	− 0.126	− 0.158
SASA				1	− 0.237*	− 0.176	− 0.336**
IPA					1	0.217*	0.465**
SIA						1	0.664**
IIA							1

Values are presented as correlation coefficients; *significant differences compared with each other at the 0.05 level; **significant differences compared with each other at the 0.01 level

The results demonstrated high validity and reliability (Table 3).

**Table 3:** The intraclass correlation coefficient (ICC)*

	AASA	PASA	SASA	SIA	IIA	IPA
Intragroup variation (A–A)	0.861	0.839	0.837	0.905	0.912	0.752
Intergroup variation (B–C)	0.823	0.812	0.809	0.878	0.895	0.736

*Values are the ICC; ICC > 0.75 indicates great reliability; ICC < 0.4 indicates poor reliability

## Discussion

Gluteal muscle contracture is a clinical disease caused by one or more pathogenic factors that induce fibrous degeneration of the gluteus muscle and its fascia, muscle contracture, and hip joint dysfunction. The evidence is inconclusive, but it is generally believed to be related to a history of repeated intramuscular injection of the Benzylalcohol during childhood [13]. The condition is most prevalent in childhood when the pelvic anatomy is not fully developed. The contracted gluteal muscles form stiff and thicker fibrous bands, comparable to tendon-like tissues, that pull and restrict normal pelvic structure development, resulting in a series of deformities when they are adult [14].

Using CT scanning to evaluate the equatorial-edge angle in GMC patients, You et al. [7] noted that it was frequently lower than normal, indicating the occurrence of acetabular retroversion in GMC patients. Reynolds et al. [15] postulated acetabular retroversion as one of the causes of hip pain in 1999. Acetabular retroversion is an anatomical variation in which the normal acetabular opening faces anterolaterally, whereas in the presence of acetabular retroversion, the opening points laterally or even posterolateral in the horizontal plane. This has been demonstrated to be an important cause of early-onset hip osteoarthritis.

The current study showed that retroversion of the acetabulum might induce by pelvic rotation as well as aberrant development of the anterior and posterior acetabular walls [9]. Furthermore, no publication on the relationship between the pelvic rotation angle and the acetabular coverage angle of gluteus contracture has been published worldwide. In this study, we found that the AASA in the GMC group increased, while the PASA decreased, indicating that the anterior margin coverage of the GMC acetabulum increased, while the posterior margin coverage decreased. At the same time, there was no significant difference in HASA between the GMC group and the control group, indicating that the GMC's overall acetabular coverage did not change significantly, but the distribution did, and this change is one of the strongest pieces of evidence of the presence of acetabular retroversion. The subsequent correlation analysis of the acetabular coverage angle and pelvic rotation angle revealed that the AASA has a significantly negative correlation with the SIA, IIA, and IPA. The PASA, on the other hand, exhibits a significantly positive correlation with the IIA. This implies that as the pelvic rotation angle decreases, the external rotation of the iliac wing increases, resulting in an increase in AASA and a decrease in PASA. This demonstrates that external rotation of the pelvic wings induces acetabular retroversion in GMC, but not abnormal development of the anterior and posterior acetabular walls.

The main muscles involved in GMC are the gluteus maximus and gluteus medius, although in severe cases, the deep-lying gluteus minimus and piriformis may be involved. The gluteus maximus is the largest muscle in the gluteal region, and it extends from the outside of the iliac wing to the gluteal crest of the femur. The gluteus medius originates on the exterior of the iliac bone between the anterior and posterior gluteal lines and ends on the lateral side of the femoral greater trochanter.

Following contracture, the muscle fibres become less elastic, stiffer, and tougher, causing the connected iliac wing to rotate backwards and outwards based on the biomechanical principle. Meanwhile, the hip bone is a composite of the ischium, iliac bone, and pubic bone. When pulled, the iliac bone in turn pulls the ischium and pubic bone below to rotate together. As a result, in this study, the pelvic rotation angles of the SIA, IIA, and IPA were all smaller than those of the control group, and the pelvic rotation angles of the SIA, IIA, and IPA in the GMC group were all positively correlated with each other and revealed statistically significant differences. The acetabulum is a broad and deep depression on the lateral side of the hip bone. At this point, the acetabulum tilts posteriorly with the posterior rotation of the hip bone, and the AASA in the GMC group increased, while the PASA decreased in comparison with the corresponding angles in the control group. Therefore, we found that the pelvic wing was wider and closer to the horizontal line through a CT cross-sectional scan in patients who were considered to have gluteal muscle contracture according to physical examination and clinical manifestations and signs. In addition, the anterior margin coverage of the GMC acetabulum increased, while the posterior margin coverage decreased. This will help us with the diagnosis of gluteus contracture and the assessment of its severity in the clinic.

Acetabular retroversion can cause wear out the acetabular labrum and articular cartilage, thereby increasing the risk of hip joint osteoarthritis [16]. By comparing the local stress on the posteriorly tilted acetabular cartilage to that with normal acetabular cartilage, Henak et al. [17] discovered that acetabular retroversion would result in a greater concentration of local stress on the acetabulum, which would in turn accelerate the progression of hip osteoarthritis. As the GMC patients included in this study were in young adulthood, imaging observations revealed no evidence of hip osteoarthritis, but this did not rule out the possibility that long-term wearing may cause joint injury.

The current treatment protocol for GMC is arthroscopic radiofrequency ablation to address the postural gait abnormalities caused by the contracture band [18]. Following the procedure, the range of motion of the hip joint is significantly increased, and the out-toeing gait is rectified. This procedure appears to have resolved the problems of GMC patients; nevertheless, it seems that the alterations in their pelvic structures did not receive adequate attention from our clinical practitioners. By assessing the above viewpoints, this study expands on the morphological alterations of the pelvis in GMC, such as external rotation of the iliac wing and acetabular retroversion. Further research is needed to determine whether these abnormal structures may be rectified after surgery and whether they hasten the development of hip osteoarthritis in GMC patients.

We acknowledge the limitations of our study. First, participants were not assessed to determine whether occupational factors had an impact on pelvic morphology. Second, the pelvic angle on only one side was measured, and it is not clear whether the same change existed on the opposite side.

## Conclusion

In this study, the rotation angle of the pelvis and acetabular coverage angle were measured by CT scans, and we found that differences of the pelvic morphology and structure existed in GMC patients compared with normal individuals. The acetabular retroversion in GMC was caused by ilial rotation rather than dysplasia of the acetabular wall.

## Data Availability

The data sets used and/or analysed in the current study are available from the corresponding author on reasonable request.

## Abbreviations

GMC: Gluteal muscle contracture
SIA: Superior iliac angle
IIA: Inferior iliac angle
IPA: Ischiopubic angle
AASA: Anterior acetabular sector angle
PASA: Posterior acetabular sector angle
HASA: Horizontal acetabular sector angle
SASA: Superior acetabular sector angle
